# Preoperative prediction of sagittal imbalance in kyphosis secondary to ankylosing spondylitis after one-level three-column osteotomy

**DOI:** 10.1186/s12891-022-05740-9

**Published:** 2022-08-18

**Authors:** Jianzhou Luo, Kai Yang, Zili Yang, Jiayi Chen, Zhengji Huang, Zhenjuan Luo, Huiren Tao, Chunguang Duan, Tailin Wu

**Affiliations:** 1 Shenzhen University Health Science Center, Shenzhen, Guangdong 518000 People’s Republic of China; 2 Department of Orthopedics, Shenzhen University General Hospital, Shenzhen, Guangdong 518000 People’s Republic of China; 3 Department of Orthopedics, Xi’an Red Cross Hospital, Xi’an, Shaanxi, 710000 People’s Republic of China; 4 Department of Neurology, the Third Affiliated Hospital of Southern Medical University, Guangzhou, Guangdong 510000 People’s Republic of China

**Keywords:** Ankylosing spondylitis, Osteotomy, Sagittal imbalance, Preoperative prediction, Optimal sagittal vertical axis

## Abstract

**Background:**

This study aimed to determine preoperative predictors for sagittal imbalance in kyphosis secondary to ankylosing spondylitis (AS) after one-level three-column osteotomy.

**Methods:**

A total of 55 patients with AS who underwent one-level three-column osteotomy were enrolled. The patients were divided into two groups according to sagittal vertical axis (SVA) value at the final follow-up (group A: SVA > 5 cm; group B: SVA ≤ 5 cm). The radiographic measures included global kyphosis, lumbar lordosis (LL), pelvic tilt (PT), pelvic incidence (PI), sacral slope, T1 pelvic angle (TPA), SVA, osteotomized vertebral angle and PI and LL mismatch (PI − LL). Postoperative clinical outcomes were evaluated using Scoliosis Research Society-22 questionnaire (SRS-22) and Oswestry Disability Index (ODI).

**Results:**

Fifty-five AS patients had an average follow-up of 30.6 ± 10.2 months (range 24–84 months). Group A had larger preoperative and postoperative LL, PT, PI − LL, TPA and SVA values compared with group B (*P* < 0.05), and no significant differences were found in ODI and SRS-22 scores between the two groups (*P* > 0.05). Preoperative LL, PT, PI − LL, TPA, and SVA values were positively correlated with the follow-up SVA value (*P* < 0.05). Among them, TPA > 40.9°, PI − LL > 32.5° and SVA > 13.7 cm were the top three predictors with the best accuracy to predict sagittal imbalance. Immediate postoperative SVA value of ≤ 7.4 cm was a key factor in reducing the risk of sagittal imbalance during follow-up.

**Conclusions:**

Preoperative TPA > 40.9°, PI − LL > 32.5° and SVA > 13.7 cm could predict sagittal imbalance in AS kyphosis after one-level three-column osteotomy, and additional osteotomies were recommended for this condition. Immediate postoperative SVA ≤ 7.4 cm was an optimal indicator for preventing sagittal imbalance.

**Level of evidence:**

IV.

## Background

Ankylosing spondylitis (AS) is a chronic inflammatory disease, which mainly affects the axial skeleton [1]. At the advanced stage, AS can be accompanied with progressively ossified spinal ligament, hyperplastic osteophytes, and rigid thoracolumbar kyphosis [1, 2]. Patients with advanced AS conditions often have trouble in standing upright, lying flat, and looking straight ahead, which seriously restricts patients’ daily activities and impairs their quality of life [3, 4]. Therefore, corrective osteotomy is often recommended for these patients to correct kyphosis and restore sagittal balance [5].

In fact, not all patients with AS who undergo osteotomy achieve a satisfactory sagittal balance, which is mainly attributed to inadequate kyphosis correction and failed postoperative sagittal realignment [6–8]. Patients with unbalanced sagittal realignment might have a poor clinical outcome, which increases the risk of pseudoarthrosis, delay union, and instrumental failure, and may even enable a second surgery [9, 10]. Thus, it is necessary to find preoperative predictions to predict sagittal imbalance in advance, and then, to determine the optimal postoperative goal for sagittal alignment construction [11, 12], thereby reducing the incidence of sagittal imbalance in AS patients. However, preoperative predictions with defined threshold values and optimal postoperative alignment of kyphosis secondary to AS following one-level three-column osteotomy have not yet been well documented. Qian et al. [9] reported that preoperative SVA and PI were predictors for sagittal imbalance. However, the thresholds of these predictors could not be figured out. Schwab et al. [10, 11, 13] suggested that postoperative SVA < 5 cm was a successful realignment but postoperative SVA > 10 cm was a failed realignment for adult spinal deformity (ASD). However, since the pathological processes of AS and ASD are different, it is unclear whether Schwab’s results are applicable to AS kyphosis correction. Therefore, preoperative predictions with clear threshold values and optimal postoperative targets for AS patients need further exploration.

In this study, we retrospectively investigated a series of patients with kyphosis secondary to AS who underwent one-level three-column osteotomy, aiming to (1) identify the difference between patients with and without sagittal imbalance; (2) figure out preoperative predictions with clear threshold values to predict sagittal imbalance; and (3) determine key factors with an optimal target for preventing sagittal imbalance.

## Methods

### Patients

Eighty-two consecutive patients with kyphosis secondary to AS who underwent three-column osteotomy from January 2011 to January 2019 were retrospectively reviewed. The inclusion criteria were as follows: 1) patients who underwent one-level three-column osteotomy; 2) patients who were followed up for a minimum of 2 years; 3) patients with complete radiographic and clinical exam data; and 4) patients with normal hip joint movement. The exclusion criteria were as follows: (1) patients with a previous history of spinal surgery; (2) patients with postoperative pseudarthrosis and instrumentation failure; and (3) patients with ankylosed hip or knee joints. Twenty cases undergoing two-level osteotomy, 2 cases with previous spinal operation history, and 5 cases with incomplete imaging or clinical data, were excluded. Finally, a total of 55 patients with AS (47 men and 8 women) who met the inclusion criteria were enrolled in this study. The average age of patients was 36.6 years (range 20–60 years), and the average follow-up duration was 30.6 months (range 24–84 months).

### Data collection

**Fig. 1:**
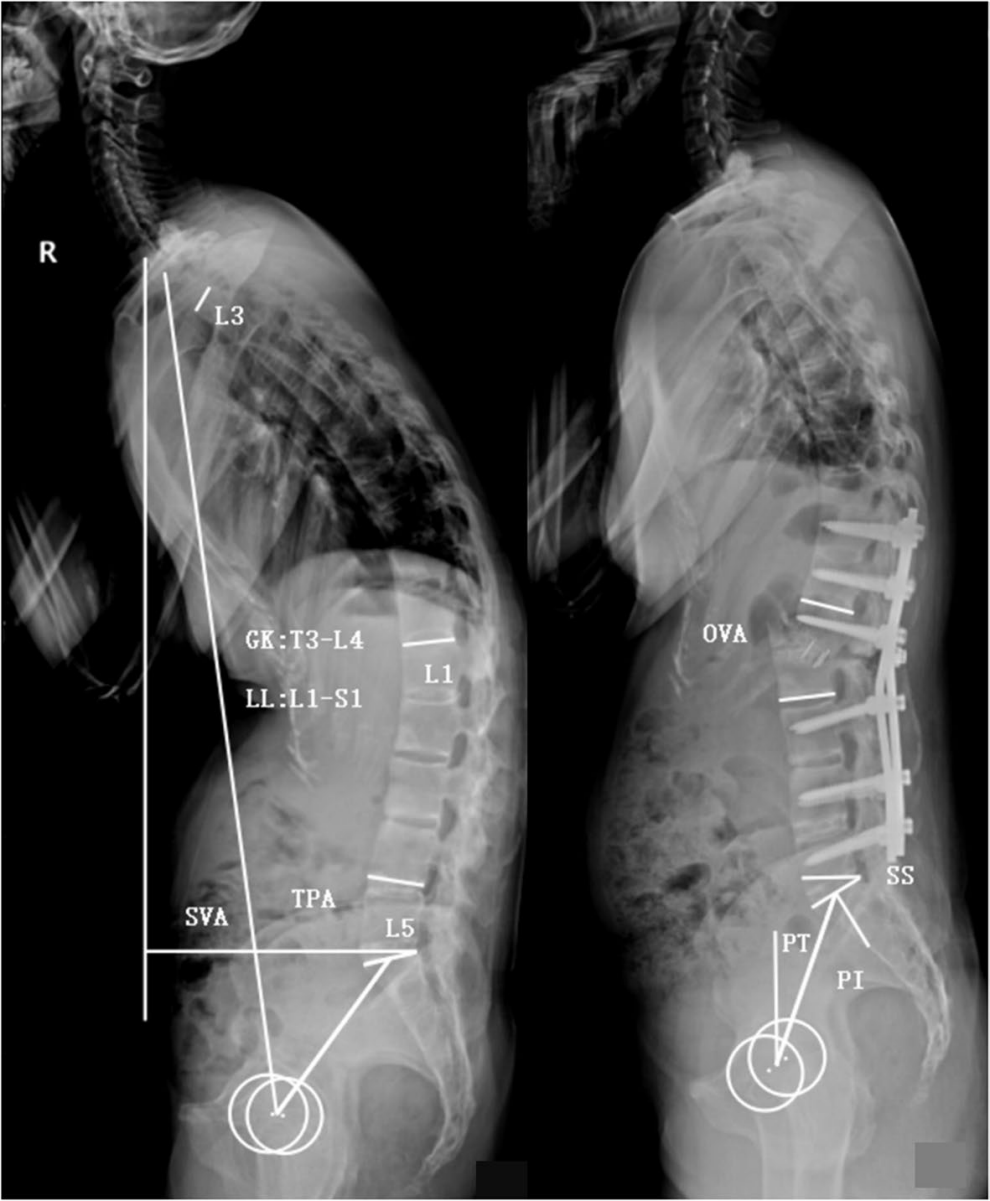
Illustration of parameters measurements. GK: the angle between the superior endplate of the maximally tilted upper-end vertebra and the inferior endplate of the maximally tilted lower-end vertebra; LL: the cobb angle from L1 upper endplate to S1 upper endplate; SVA, the distance between the C7 plumb line and the posterior–superior corner of S1; TPA: the angle between the line from the center of T1 vertebral body to the center of femoral head axis and the line from the center of S1 upper endplate to the center of femoral head axis; PT: the angle between the vertical line and the line from the center of S1 upper endplate to the center of femoral head axis; PI: the angle between the perpendicular line to the S1 upper endplate and the line from the center of S1 upper endplate to the center of femoral head axis; SS: the angle between S1 upper endplate and the horizontal line; OVA: the angle between the lower end plate of the osteotomized vertebra and the upper end plate of the cranial adjacent vertebra

Anteroposterior and lateral radiographs of the whole spine while standing were obtained preoperatively, postoperatively, and at the final follow-up. The following parameters were measured using lateral radiographs: global kyphosis (GK), lumbar lordosis (LL), T1 pelvic angle (TPA), pelvic tilt (PT), PI, sacral slope (SS), osteotomized vertebral angle (OVA) and PI and LL mismatch (PI − LL) (Fig. 1). The clinical outcomes were evaluated using the Scoliosis Research Society-22 (SRS-22) questionnaire and Oswestry Disability Index (ODI). The patient with a follow-up SVA of > 5 cm was regarded as sagittal imbalance [10, 14].

### Surgical technique

A modified pedicle subtraction osteotomy was performed in the apical region of kyphosis, and somatosensory- and motor-evoked potentials were monitored throughout the procedure.

After general anesthesia, the pedicle screws were implanted at the planned levels of fusion. The resection area included the spinous process, upper part of the lamina, and superior articular processes of the osteotomized vertebra, as well as the lower part of the lamina and inferior articular processes of the cranial adjacent vertebra. The transverse process of the osteotomized vertebra was exposed and resected. Subsequently, subtotal resection was performed along the upper part of the pedicle to the front of the vertebral body, usually allowing removing the upper one third to half of the vertebral body together with the adjacent intervertebral disk in the skull. Following osteotomy, bilateral temporary rods were implanted firmly on at least two vertebrae above and below the osteotomized site. During the correction, the surgeons slightly closed both sides of the osteotomized ends by compressing the rods to slightly shorten the spinal cord in advance because the spinal cord tended to elongate during correction. Then, the circuit nurse and technician lifted the patient’s shoulders and gradually removed the postural pads to correct kyphosis. Simultaneously, the surgeons used the point of the rods at the osteotomized gap as a hinge and bent it while the patient’s shoulders were still lifted to restore spinal realignment. After achieving satisfactory correction, the temporary rods were replaced with precontoured rods successively. Subsequently, a local bone graft and a cage filled with autogenous bone were implanted into the osteotomy space, further compressing the rods. The bone autograft was paved on the surface of the lamina to facilitate spinal fusion.

### Statistical analysis

Statistical analysis was performed using SPSS software (version 22.0, SPSS Inc., IL, USA). All numeric parameters were expressed as mean ± standard deviation (SD). The differences in quantitative data between the two groups were compared using independent-samples *t*-test. Qualitative data were analyzed by *χ*^2^ test or Fisher’s exact test. The correlations between SVA and preoperative/postoperative parameters were analyzed with Pearson correlation coefficients. Receiver-operating characteristic (ROC) analysis was performed to determine the preoperative predictors and to calculate their thresholds with maximum Youden index. Logistic regression analysis was performed to determine the key postoperative parameter, and the threshold of this key parameter was evaluated using the ROC and the maximum Youden index. A *P* value < 0.05 was considered statistically significant.

## Results

### Clinical and radiographic data

The follow-up SVA was > 5 cm in 37 patients in group A and was ≤ 5 cm in 18 patients in group B after a 2-year follow-up. These two groups were well balanced with regard to average age, sex distribution, and osteotomy sites (*P* > 0.05, Table 1); and the operative time, blood loss and fusion levels were also similar (*P* > 0.05, Table 1). Group A patients had larger preoperative and postoperative LL, PT, PI − LL, TPA, and SVA values compared with group B patients (*P* < 0.05, Table 2). Both groups achieved similar kyphosis corrections postoperatively (*P* > 0.05, Table 2). No significant difference was found in GK, PI, SS, OVA, ODI score and SRS-22 score between these two groups (*P* > 0.05, Table 2).

**Table 1:** Comparison of demographic and surgical data between two groups

Variables	Group A (*n* = 37)	Group B (*n* = 18)	*P* value
Age (year)	38.3 ± 8.2	34.9 ± 8.1	0.152
Sex (M/F)	33/4	14/4	0.472
Operative time (min)	333.1 ± 91.6	331.8 ± 58.2	0.956
Blood loss (ml)	1145.1 ± 871.9	1673.5 ± 1092.9	0.062
Osteotomy sites (n)			
T12	1	1	0.752
L1	8	5	
L2	22	8	
L3	6	4	
OVA (°)	39.5 ± 13.4	33.5 ± 11.3	0.110
Fusion level (n)	6.3 ± 1.1	6.5 ± 1.7	0.709
Follow-up (month)	29.1 ± 4.0	32.2 ± 3.9	0.862

*OVA* Osteotomized vertebral angle

**Table 2:** Differences of radiographic and clinical measurements between group A and B

Measurements	Group A (*n* = 37)	Group B (*n* = 18)	*P* value
Pre-GK (°)	79.4 ± 21.7	74.7 ± 16.3	0.425
Post-GK (°)	35.0 ± 14.4	36.4 ± 18.8	0.769
Correction-GK (°)	43.8 ± 14.2	38.3 ± 14.9	0.198
Pre-LL (°)	8.1 ± 21.4	-7.3 ± 16.6	0.010^*^
Post-LL (°)	-30.5 ± 14.6	-41.5 ± 18.9	0.020^*^
Correction-LL (°)	38.5 ± 14.1	34.3 ± 18.8	0.354
Pre-PT (°)	41.0 ± 11.1	34.1 ± 8.4	0.024^*^
Post-PT (°)	30.9 ± 9.9	23.5 ± 9.7	0.011^*^
Correction-PT (°)	10.0 ± 10.0	10.7 ± 8.3	0.820
Pre-PI (°)	48.5 ± 13.4	44.9 ± 14.6	0.374
Post-PI (°)	48.9 ± 12.3	45.3 ± 12.2	0.313
Correction-PI (°)	0.5 ± 6.9	0.4 ± 6.5	0.962
Pre-SS (°)	7.5 ± 12.6	10.8 ± 13.1	0.374
Post-SS (°)	18.0 ± 10.2	21.9 ± 14.6	0.256
Correction-SS (°)	10.5 ± 9.2	11.0 ± 9.2	0.837
Pre-PI-LL (°)	56.5 ± 18.5	37.6 ± 20.3	0.001^*^
Post-PI-LL (°)	18.4 ± 15.2	3.7 ± 11.4	< 0.001^*^
Correction-PI-LL (°)	38.1 ± 14.0	33.9 ± 20.4	0.376
Pre-TPA (°)	58.6 ± 15.9	42.2 ± 14.6	0.001^*^
Post-TPA (°)	23.2 ± 7.1	16.3 ± 8.6	< 0.001^*^
Correction-TPA (°)	25.3 ± 12.0	22.2 ± 13.2	0.399
Pre-SVA (cm)	23.2 ± 7.1	16.3 ± 8.6	0.002^*^
Post-SVA (cm)	10.9 ± 3.6	3.6 ± 3.1	< 0.001^*^
Correction-SVA (cm)	12.5 ± 5.9	12.6 ± 9.4	0.959
Pre-ODI score	38.46 ± 20.85	43.15 ± 18.84	0.443
Post-ODI score	25.75 ± 14.74	18.65 ± 10.40	0.087
Correction- ODI score	13.30 ± 23.32	24.50 ± 20.74	0.105
Pre-SRS22 score	2.63 ± 0.69	2.91 ± 0.50	0.277
Post-SRS22 score	3.95 ± 0.49	4.05 ± 0.62	0.067
Correction- SRS22 score	1.32 ± 0.77	1.14 ± 0.68	0.758

Negative number represents lordosis, positive number represents kyphosis

*GK* Global kyphosis, *LL* Lumbar lordosis, *PT* Pelvic tilt, *PI* Pelvic incidence, *SS* Sacral slope, *PI-LL* PI minus LL value, *TPA* T1 pelvic angle, *SVA* Sagittal vertical axis, *SRS-22* Scoliosis Research Society-22 questionnaire, *ODI* Oswestry Disability Index

^*^The difference between group A and B was statistically significant (*P* < 0.05)

### Preoperative predictions for sagittal imbalance

**Fig. 2:**
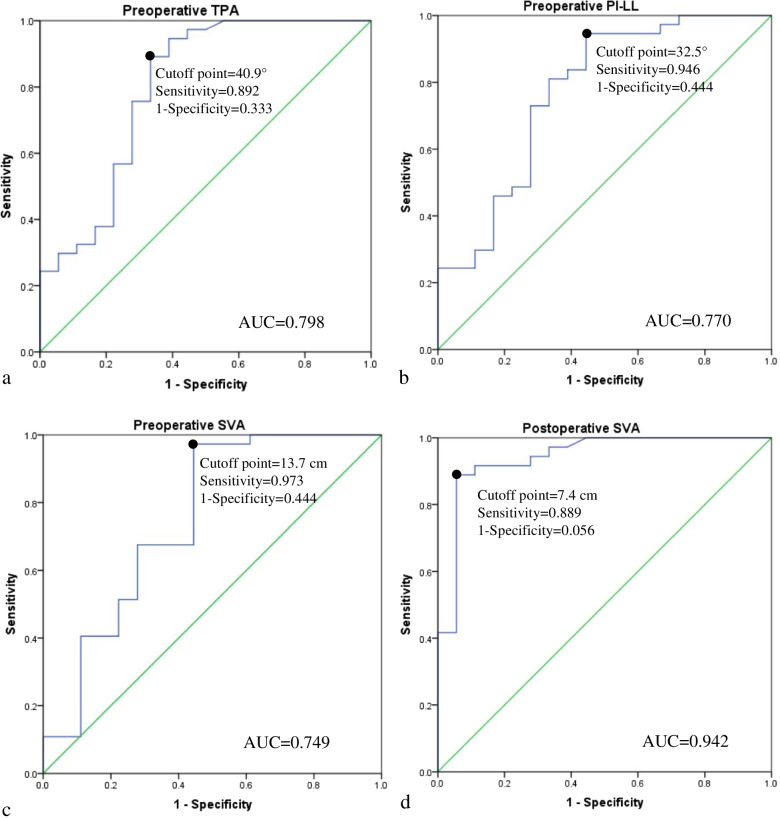
Receiver-operating characteristic (ROC) curve for determining the cutoff point of the preoperative TPA (**a**), preoperative PI-LL (**b**), preoperative SVA (**c**) and postoperative SVA (**d**), with an area under the curve (AUC), a sensitivity and a false-positive rate (1-specificity). TPA, T1 pelvic angle; PI-LL, PI minus LL value; SVA, sagittal vertical axis

The correlation analyses between the follow-up SVA and preoperative parameters demonstrated that preoperative LL, PT, PI − LL, TPA, and SVA values were positively correlated with the follow-up SVA value (*P* < 0.05, Table 3). Based on ROC analysis, the area under the ROC curve (AUC) of LL, PT, PI − LL, TPA and SVA was 0.712, 0.700, 0.770, 0.798, and 0.749, respectively. The top three preoperative parameters (TPA, PI − LL and SVA) with the highest AUC value were regarded as the predictions for postoperative sagittal imbalance. The prediction with TPA > 40.9° yielded a sensitivity of 89.2% and a false-positive rate (1-specificity) of 33.3% (Fig. 2a); PI − LL > 32.5° prediction had a sensitivity of 94.6% and a false-positive rate of 44.4% (Fig. 2b); SVA > 13.7 cm was associated with a predictive sensitivity of 97.3% and a false-positive rate of 44.4% (Fig. 2c).

**Table 3:** Correlations between the follow-up SVA and the pre-/postoperative parameters

Parameters	Coefficient, r	*P* value
Preoperative GK	0.124	0.367
Preoperative LL	0.281	0.038^*^
Preoperative PT	0.392	0.003^*^
Preoperative PI	0.233	0.087
Preoperative SS	-0.078	0.574
Preoperative PI-LL	0.437	0.001^*^
Preoperative TPA	0.454	< 0.001^*^
Preoperative SVA	0.386	0.004^*^
Postoperative GK	0.039	0.781
Postoperative LL	0.389	0.003^*^
Postoperative PT	0.437	0.001^*^
Postoperative PI	0.218	0.111
Postoperative SS	-0.159	0.245
Postoperative PI-LL	0.661	< 0.001^*^
Postoperative TPA	0.669	< 0.001^*^
Postoperative SVA	0.834	< 0.001^*^

*GK* Global kyphosis, *LL* Lumbar lordosis, *PT* Pelvic tilt, *PI* Pelvic incidence, *SS* Sacral slope, *PI-LL* PI minus LL value, *TPA* T1 pelvic angle, *SVA* Sagittal vertical axis

^*^ Indicated that the correlation was statistically significant (*P* < 0.05)

**Fig. 3:**
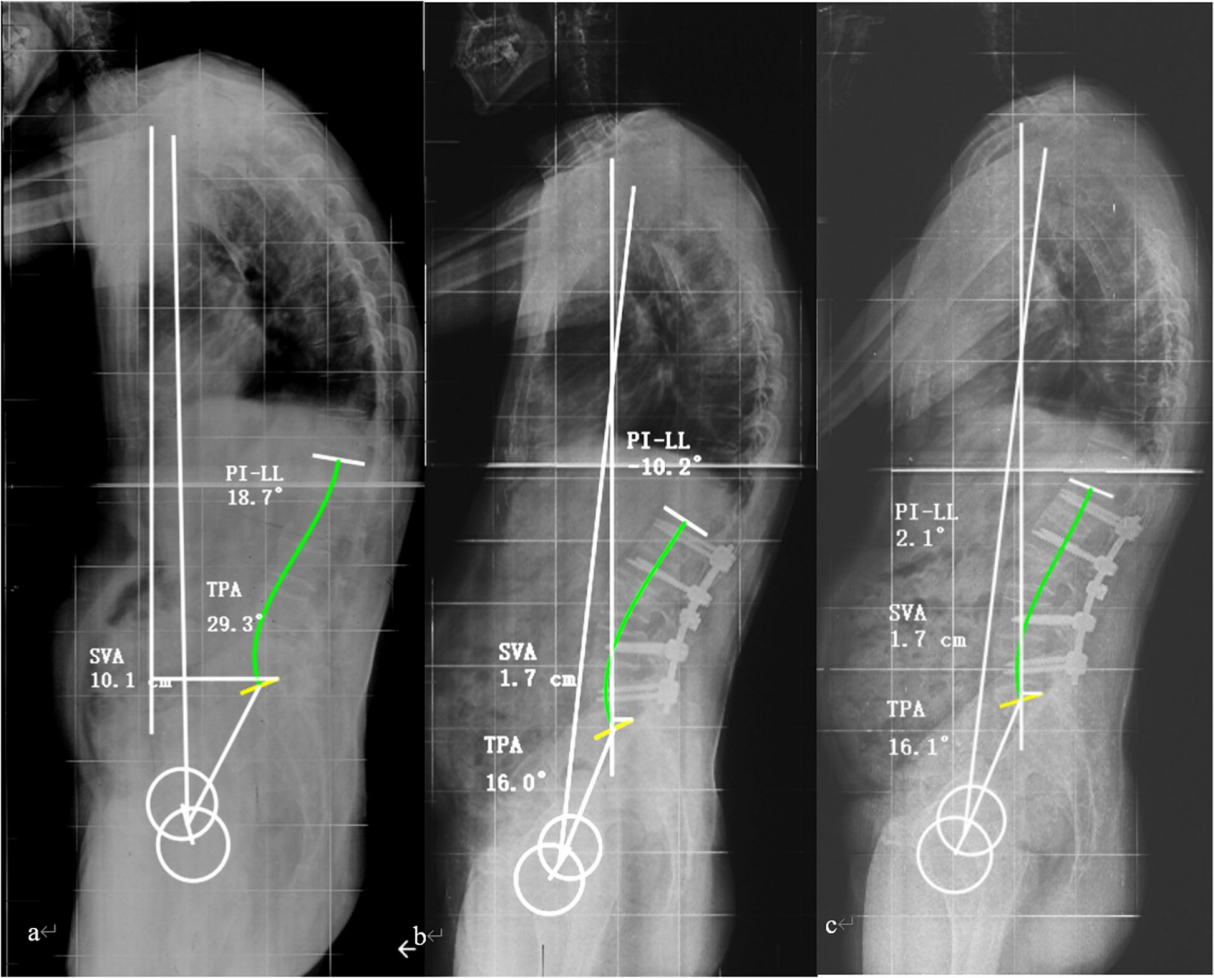
A 46-year-old man developed thoracolumbar kyphosis secondary to AS for 15 years. **a** Preoperatively, the patient presented a thoracolumbar kyphosis and sagittal imbalance with TPA = 29.3° (< 40.9°), PI – LL = 18.7° (< 32.5°), and SVA = 10.1 cm (< 13.7 cm), who met no threshold value of the preoperative predictors and was predicted a good sagittal realignment after one-level 3-column osteotomy; **b** After an osteotomy on L3, the kyphosis was corrected and the sagittal alignment was restored properly with the SVA of 1.7 cm (< 7.4 cm), which was less than the optimal postoperative SVA; **c** At the follow-up of 33 months, the patient displayed a maintained correction and a good sagittal alignment with the SVA of 1.7 cm (sagittal balance), which was consistent with the result of the prediction with preoperative predictors

**Fig. 4:**
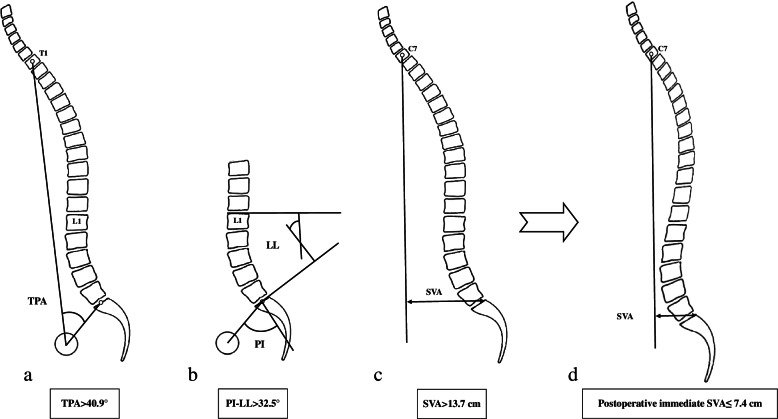
Schematic diagram of preoperative prediction for sagittal imbalance. **a**-**c** Two or three preoperative parameters met the thresholds of TPA > 40.9°, PI—LL > 32.5° and SVA > 13.7 cm, a high risk of sagittal imbalance was predicted following one-level three column-osteotomy, and additional osteotomies was recommended. **d** The goal for preventing postoperative sagittal imbalance was to reconstruct the immediate postoperative SVA of ≤ 7.4 cm

The predictive accuracy of these preoperative parameters with clear thresholds was verified in this cohort. The results showed that patients with two or three of these predictors reaching the threshold values had a significantly high risk of sagittal imbalance during follow-up. Patients with only one predictor or none of the predictors meeting the thresholds experienced a low risk of sagittal imbalance during follow-up (Table 4, Figs. 3 and 4).

**Table 4:** Efficacy of these preoperative predictors with the threshold values to predict sagittal imbalance

Numbers of predictors met the thresholds	Cases	Sagittal balance	Sagittal imbalance	*P* value
No predictor met	9	9	0	/
One predictor met	3	2	1	0.250
Two predictors met	7	1	6	0.001^*^
All predictors met	36	6	30	< 0.001^*^

^*^ Compared with “No predictor met”, the difference was statistically significant (*p* < 0.05)

### Key factor for preventing sagittal imbalance

The immediate postoperative PI − LL, TPA and SVA values were the top three relevant parameters with the highest coefficient to sagittal imbalance at the follow-up, so they were entered into the logistic regression analysis. The results revealed that postoperative SVA value was an independent factor for preventing sagittal imbalance (*P* < 0.001, Table 5). ROC analysis of postoperative SVA demonstrated that SVA ≤ 7.4 cm for preventing sagittal imbalance during follow-up had an AUC of 0.941, with a sensitivity of 88.9%, and a false-positive rate of 5.6% (Fig. 2d). The patients with an immediate postoperative SVA of ≤ 7.4 cm had a smaller SVA and developed a lower incidence of sagittal imbalance during follow-up compared with those with an immediate postoperative SVA > 7.4 cm (*P* < 0.001, Table 6). There were no significant differences in ODI and SRS-22 scores between the two groups (*P* > 0.05, Table 6) (Fig. 4).

**Table 5:** Logistic regression analysis of the postoperative parameters for sagittal imbalance

Variable	B	SE	Wald	*P* value	Exp(B)	95%CI of Exp(B)	
						**Lower**	**Upper**
Postoperative SVA	0.680	0.192	12.555	< 0.001	1.975	1.355	2.877
**(Constant)**	-4.054	1.322	9.403	0.002	0.017		

With the follow-up sagittal imbalance as dependent variable; SVA, sagittal vertical axis

**Table 6:** Efficacy of immediate postoperative SVA to sagittal imbalance at the follow-up

**Variable**	**Above threshold value** **(> 7.4 cm)**	**Below threshold value** **(**≤ **7.4 cm)**	***P*** **value**
Cases (n)	34	21	/
Postoperative SVA (cm)	11.5 ± 3.1	3.6 ± 2.4	< 0.001^*^
Follow-up SVA (cm)	11.4 ± 3.4	4.1 ± 3.3	< 0.001^*^
Incidence of sagittal imbalance at the follow-up	97.1% (33/34)	19.0% (4/21)	< 0.001^*^
Follow-up ODI score	24.11 ± 14.75	21.28 ± 13.16	0.553
Follow-up SRS-22 score	3.89 ± 0.50	4.04 ± 0.51	0.382

*SVA* Sagittal vertical axis, *SRS-22* Scoliosis Research Society-22 questionnaire, *ODI* Oswestry Disability Index

^*^ Indicated that the difference was statistically significant between groups (*P* < 0.05)

### Complications

Sixteen postoperative complications were noted in 11 patients, including 1 pleural effusion, 4 transient neurological damage, 5 vertebral subluxation and 6 dural tear (all of them with dural tear complicated with Andersson lesions at the osteotomized sites). No screw loosening, rod breakage and pseudoarthrosis were observed at the final follow-up.

## Discussion

Osteotomy is an effective method to correct kyphosis and restore the sagittal alignment in patients with AS, which greatly improves their daily living activities and quality of life [5, 9, 12, 15]. However, clinically, not all patients with kyphosis who undergo osteotomy can achieve a satisfactory sagittal balance during follow-up, leading to an increased risk of implant failure, delayed union, pseudoarthrosis, and correction loss [8, 16]. Until now, few studies have attempted to specifically determine the preoperative predictors with defined threshold values to predict sagittal imbalance in AS patients, and the optimal postoperative targets for preventing sagittal imbalance in these patients are still less known [9, 12, 13].

In this study, the patients in group A had larger preoperative LL, PI − LL, TPA, and SVA values than those in group B, which indicated that the patients who developed sagittal imbalance during follow-up often had more severe preoperative sagittal imbalance and spinopelvic deformity. These patients were supposed to undergo a matching larger correction to construct their sagittal alignment with the apparently preoperative sagittal deformity. However, in fact, they only received a similar magnitude of corrections as those in group B, leaving much residual postoperative deformity. As a result, patients in group A were more likely to experience failed sagittal realignment than those in group B during follow-up. This finding was consistent with that observed by Schwab et al. [13], who indicated that one-level three-column osteotomy might not always achieve a satisfactory outcome, particularly for those with severe preoperative sagittal imbalance and lumber kyphosis. Therefore, additional osteotomy was recommended for those patients [13, 17].

Pearson coefficient analysis demonstrated that the follow-up SAV value was significantly corelated with preoperative parameters, which made it possible to predict sagittal imbalance with preoperative parameters at the final follow-up. Based on the significant correlations between the follow-up SVA value and preoperative parameters, ROC analysis showed that TPA, PI − LL, and SVA prediction had the top three AUC values, which indicated that these three preoperative parameters were the optimal predictors with the best accuracy for sagittal imbalance, with the optimal thresholds of TPA > 40.9°, PI − LL > 32.5°, and SVA > 13.7 cm, respectively. Similarly, Qian et al. [9] found that the SVA and PI were radiographic predictors for postoperative sagittal imbalance; however, the optimal threshold value of these two predictors could not be determined. In the present study, the three selected preoperative predictions for sagittal imbalance had the largest AUC; and their threshold values were well-defined, making them useful in surgical decision-making. Although preoperative LL and PT values showed a statistical relevance with sagittal imbalance, the accuracy and coefficient were relatively lower compared with the other three parameters; thus, they were not included in the subsequent analyses. Additionally, the predictive accuracy of these three factors were validated in the present cohort. The results showed that patients with two or three predictors meeting the thresholds had significantly increased risk of sagittal imbalance at the final follow-up. This finding revealed that higher predictive ability was achieved when using two or three predictors reaching thresholds concurrently. Only using one factor meeting the threshold value might not be enough to accurately predict sagittal imbalance. Therefore, based on these results, two or three preoperative predictors were recommended to be taken into consideration concurrently when performing preoperative planning and judging the postoperative sagittal realignment.

Predicting sagittal realignment in advance was the first step for preventing sagittal imbalance, while correcting kyphosis and constructing sagittal alignment should be the most important steps. The main cause for failed sagittal realignment in most patients has always been attributed to inadequate intraoperative correction [13, 18]. Schwab et al. [11] suggested that the postoperative SVA value should be < 4.7 cm for ASD after osteotomy. However, for kyphosis in patients with AS, the optimal postoperative SVA value for correction is still unclear. In this study, based on analyzing the relationship between the follow-up SVA and postoperative parameters, we investigated the key parameter for sagittal imbalance with logistic regression analysis. The top three postoperative parameters (PI − LL, TPA, and SVA) with the highest coefficient were entered into logistic regression analysis to determine the independent key parameter of sagittal imbalance at the final follow-up. The result demonstrated that the immediate postoperative SVA was the independent key factor for sagittal imbalance. Moreover, the optimal threshold of postoperative SVA for preventing sagittal imbalance was ≤ 7.4 cm, with an AUC of 0.941, a sensitivity of 88.9%, and a false-positive rate of 5.6%.

Further comparison also verified that patients with an immediate postoperative SVA ≤ 7.4 cm had a smaller SVA and a lower incidence of sagittal imbalance than those with postoperative SVA of > 7.4 cm. This result was in line with that by Kim et al. [8] who reported that restoration and maintenance of postoperative SVA < 8 cm was important for ultimate sagittal reconstruction in fixed sagittal imbalance. In our study, the patients with an immediate postoperative SVA of ≤ 7.4 cm maintained an acceptable sagittal realignment during follow-up. Similarly, Wang et al. [19] also reported that AS patients with a postoperative SVA of 8.6 cm usually did not show an obvious correction loss or severe sagittal imbalance during follow-up. Kim and his colleague [5] observed 248 AS patients and found that the patients with a SVA of 7.0 cm or less achieved the best clinical outcome after surgery. These results commonly reminded that for AS patients with severe kyphosis, the SVA would not be required to be corrected to a normal range, and the postoperative SVA of ≤ 7.4 cm might be enough for most patients to achieve satisfied clinical outcomes.

Interestingly, AS patients with a relatively larger immediate postoperative SVA might partially reduce their SVA to a smaller range during follow-up. In general, the spine of advanced AS patients was stiff and rigid, and could not be flexed and extended. However, the pelvis functioned as a compensatory mechanism with the femoral heads as the fulcrum to rotate anteriorly after surgery. As the pelvis was rotated backward to compensate sagittal imbalance preoperatively, the posteriorly rotated pelvis could have some degree of anterior rotation postoperatively in the absence of hip stiffness. Of note, the pelvis forward rotation could not be accomplished immediately after surgery, because AS with severe pelvic retroversion was usually complicated with hip joint contracture caused by ligament tension, which could be gradually corrected to a certain extent with rehabilitation exercises [4, 20, 21]. In addition, AS patients were still accustomed to the preoperative anterior center of gravity; thus, they kept flexing the trunk while standing until they got used to the new center of gravity after a period of time.

Of course, there were dynamic changes of postoperative sagittal parameters because of wound pain, gravity adjustment, muscle strength and so on [22]. However, it was reported that the sagittal parameters were largely stable one month postoperatively [22–24]. Thus, we chose the postoperative 3–4 week as the postoperative immediate period and measured radiographic parameters. At this time point, the wound pain was basically recovered, and patients adapted to the adjustment of the center of gravity and were able to take X-rays upright alone.

Advanced AS were usually complicated with osteoporosis, which might also increase the risk of follow-up sagittal imbalance [25, 26]. The holding force of pedicle screws in bones with low mineral density were small and weak, easily leading to screw loosening, nail extraction, and delayed union or nonunion at osteotomy sites [27, 28]. Consequently, the internal fixation failure occurred, resulting in sagittal imbalance during the mid- and long-term follow-up. Therefore, all AS patients undergoing osteotomy in our study received a routine osteoporosis management before and after surgery, including the treatments with vitamin D, calcium, bisphosphonates and RANKL monoclonal antibodies [29]. Owing to the osteoporosis management, only one proximal junctional kyphosis and no bone graft subsidence occurred in this cohort, so the incidence of complications was lower than those reported in previous studies [7, 19]. Besides, implanting a cage in the osteotomized gap might also contribute to the low subsidence rate of the osteotomized ends. All AS patients was routinely required to wear a thoracolumbar brace for at least 3 months postoperatively, which might also account for the low rate of bone subsidence and junctional disease.

This study has some limitations. First, although the choices of osteotomized sites and the degree of correction might influence sagittal realignment, they were not compared separately because of the limited sample size. Second, the hip flexibility of these patients was not quantified, which might influence the recovery of SVA postoperatively. Third, the patients with a postoperative SVA ≤ 7.4 cm still had 19.0% incidence of sagittal imbalance, and further analysis with a larger sample size was needed to obtain a precise SVA threshold. Fourth, some AS patients retained some degree of spinal flexibility, which might contribute to the recovery of sagittal balance postoperatively. However, few studies have explored this effect and further study are warranted.

## Conclusions

AS patients with larger preoperative sagittal alignment were more likely to experience sagittal imbalance postoperatively. Preoperative TPA > 40.9°, PI − LL > 32.5° and SVA > 13.7 cm could predict sagittal imbalance during follow-up, and additional osteotomies were recommended for this condition. Immediate postoperative SVA ≤ 7.4 cm was an alignment for preventing sagittal imbalance.

## Data Availability

The patients’ data were collected in the Shenzhen University General hospital. The datasets used and/or analyzed during the current study are available from the corresponding author on reasonable request.

## Abbreviations

AS: Ankylosing spondylitis
ASD: Adult spinal deformity
GK: Global kyphosis
LL: Lumbar lordosis
ODI: Oswestry Disability Index
OVA: Osteotomized vertebral angle
PI: Pelvic incidence
PI-LL: PI and LL mismatch
PT: Pelvic tilt
SRS-22: Scoliosis Research Society-22 questionnaire
SS: Sacral slope
SVA: Sagittal vertical axis
TPA: T1 pelvic angle
